# Correlates of physical activity among rural Australian cancer survivors: a cross-sectional study

**DOI:** 10.1007/s00520-025-09598-0

**Published:** 2025-06-02

**Authors:** Michael James Leach, Stephanie Monacella, Thi Trinh, Anny Byrne, Eli Ristevski

**Affiliations:** 1 School of Rural Health, Monash University, Bendigo, VIC Australia; 2 West Gippsland Healthcare Group, Warragul, VIC Australia; 3 Peninsula Health, Frankston, VIC Australia; 4 School of Rural Health, Monash University, Warragul, VIC Australia

**Keywords:** Physical activity, Obesity, Fatigue, Cancer survivors, Rural

## Abstract

**Purpose:**

This study aimed to identify correlates of physical activity (PA) among rural Australian cancer survivors.

**Methods:**

A cross-sectional study was undertaken. We recruited a convenience sample of adults diagnosed with any cancer type who received care at a rural hospital in Gippsland, Victoria, Australia, between August 2017 and December 2021. PA was measured using the Godin–Shephard Leisure-Time PA Questionnaire (GSLTPAQ). Correlates of sufficient PA (total GSLTPAQ score ≥ 14), any mild PA (item-specific GSLTPAQ score > 0), and any PA (item-specific GSLTPAQ score > 0 for one or more intensities: mild, moderate and/or strenuous) were identified via binary logistic regression.

**Results:**

There were 103 participants with a median age of 66 years. Among them, 35% had sufficient PA, 62% had any mild PA, and 64% had any PA. Obesity was associated with lower odds of sufficient PA (adjusted odds ratio (aOR) = 0.21, 95% confidence interval (CI) = 0.07–0.61), any mild PA (aOR = 0.26, 95% CI = 0.10–0.66), and any PA (aOR = 0.26, 95% CI = 0.10–0.64). Fatigue was associated with lower odds of sufficient PA (aOR = 0.30, 95% CI = 0.11–0.83) but not with any mild PA or any PA. Breast cancer survivors had lower odds of any mild PA (aOR = 0.27, 95% CI = 0.08–0.87) but not of any PA or sufficient PA.

**Conclusion:**

This study identified levels and correlates of PA among rural Australian cancer survivors. While further research is warranted, our results suggest considerable scope for exercise oncology interventions in a rural region of Australia. Exercise interventions for cancer survivors in this and similar rural settings could be tailored to and targeted towards those with obesity, fatigue, and/or breast cancer.

## Introduction

The global incidence of cancer has been estimated at 20 million new cases for the year 2022, and this is projected to increase to 35 million new cases by 2050 [1]. Cancer adversely affects both quantity and quality of life [1–3]. People diagnosed with cancer (henceforth termed cancer survivors) experience adverse effects triggered by their cancer type and treatment modality, including but not limited to pain, peripheral neuropathy, sexual dysfunction, fatigue, mental health problems, and heart failure [2, 3].

Exercise is a structured approach to increasing physical activity (PA) that has emerged as a non-invasive, nonpharmacological approach to cancer care, and that is supported by a growing body of exercise oncology literature [4, 5]. Exercise has been shown to lower the risk of treatment-induced adverse effects, cancer recurrence, and mortality, and to improve overall and domain-specific health-related quality of life (HRQoL), among cancer survivors [4, 5]. For example, exercise may reduce the risk of chemotherapy-induced cardiac impairment among breast cancer survivors, thereby potentially protecting against heart failure [6]. Peak bodies (e.g., American Society of Clinical Oncology and Clinical Oncology Society of Australia) recommend exercise for cancer survivors [7, 8], while health services and other organisations (e.g., Cancer Council Western Australia) have integrated exercise programs into routine cancer care [9, 10]. Cancer survivors with low PA levels, and for whom exercise is feasible and not contraindicated, are likely to be in relatively high need of increasing PA through exercise.

Internationally, subgroups of cancer survivors with insufficient PA have been identified in quantitative studies exploring demographic, psychosocial, clinical, and energy-level factors associated with PA measured via questionnaire or accelerometer [11–29]. Most of these studies were conducted among breast cancer survivors only or, alternatively, among survivors of multiple unspecified cancer types [11, 16–22, 26, 29]. Few studies measured PA using different intensity outcomes (e.g., mild, moderate and strenuous PA), which permit a more nuanced assessment of PA [14, 18, 24, 26, 29]. Importantly, few of these studies were conducted among rural—as opposed to metropolitan—cancer survivors, and all such rural studies were conducted in North America [12, 25–28]. One of the rural studies had a limitation whereby only bivariate analysis was undertaken [12]. In this past rural study, effect estimates were not adjusted for potential confounding factors, potentially due to the lower sample size of 72 [12].

It is important to assess correlates of PA among rural cancer survivors. This is, firstly, because studies suggest rural residents have PA levels that are worse or no better than their metropolitan counterparts—both in the general population and among cancer survivors [30–32]. Evidence also suggests that, compared with the general population, rural and metropolitan cancer survivors have clinically lower physical HRQoL—a factor related to PA [33]. Moreover, rural residents experience barriers to accessing health care, while rural health services tend to be under-resourced relative to metropolitan health services and may also have poor resource management [34, 35]. Further studies investigating correlates of PA among rural cancer survivors could help to inform localised models of cancer care by, firstly, identifying subgroups of rural cancer survivors who are in greatest need of exercise interventions (e.g., health coaching by an exercise physiologist [9]). Some of the limited resources available to rural health services could then be targeted towards supporting those rural cancer survivors with relatively low PA, thereby potentially enhancing the efficiency of rural cancer service delivery.

No known studies have explored demographic, psychosocial, clinical, and/or energy-level factors associated with PA among rural cancer survivors in Australia. We, therefore, aimed to identify correlates of PA among rural Australian cancer survivors diagnosed with any cancer type. We had two research questions: (1) What patient characteristics are associated with meeting PA guidelines among rural Australian cancer survivors? (2) What patient characteristics are associated with engaging in PA of different intensities among rural Australian cancer survivors?

## Methods

### Setting

The present study was set in Gippsland—a region in rural Victoria, Australia, with a population of 154,357 [36]. Compared with the rest of the state of Victoria, Gippsland’s population is older (median age, 46 cf. 39 years) and has higher rates of potentially preventable diseases [36].

### Design and sampling

A cross-sectional study design was employed. We used baseline (pre-intervention) data from a prospective cohort study of a health coaching intervention for rural cancer survivors—the I.CAN Program [9]. The study population was a convenient sample of people diagnosed with cancer who participated in I.CAN, which is offered as part of routine cancer care at a rural hospital in Gippsland. Recruitment took place between August 2017 and December 2021. Participants were included if they were aged 18+ years and had a diagnosis of any cancer type, but excluded if they experienced acute malnutrition or received end-of-life care. The study population was not restricted to those on active anti-cancer treatment because, in accordance with the National Coalition for Cancer Survivorship’s definition, we defined cancer survivors as anyone diagnosed with cancer [37].

### Data collection

For each participant, all baseline data were collected on the same date. These data were collected from participants and hospital medical records using paper-based forms. Data were securely entered and stored in a password-protected Microsoft Access database (Microsoft Corp., Redmond, WA, USA).

### Physical activity measures

The Godin–Shephard Leisure-Time Physical Activity Questionnaire (GSLTPAQ) was used to measure PA [38]. The GSLTPAQ is a commonly used, valid, and reliable PA measure that has been validated for use in cancer survivors, through assessing associations between GSLTPAQ scores and PA variables measured via accelerometers/pedometers [39]. The GSLTPAQ measures an important PA subtype called leisure-time physical activity (LTPA)—PA undertaken in one’s own time [38]. As cancer is primarily a disease of the aged, LTPA likely represents a greater proportion of the average cancer survivor’s PA than the PA of the average person in the general population. The GSLTPAQ includes a three-part question on frequencies with which respondents engaged in 15+ min of mild, moderate, and strenuous PA in their own time throughout a typical week [38]. In the GSLTPAQ, mild PA is PA requiring minimal effort (e.g., yoga and easy walking), moderate PA is PA that requires more than minimal effort without being exhausting (e.g., dancing and brisk walking), and strenuous PA is PA that makes one’s heart beat rapidly (e.g., basketball and running) [38]. The total GSLTPAQ score—the Leisure Score Index (LSI)—is calculated by multiplying the weekly frequency for each of the mild, moderate, and strenuous PA items by three, five, and nine metabolic equivalents, respectively, before summing the three individual results to give a total number [40]. In our study, one of the three outcomes of interest was a binary variable categorising participants in terms of whether or not they had sufficient PA measured via the LSI. Participants were categorised as sufficiently physically active if their LSI score was 14+ (the non-reference group) and insufficiently active if their LSI score was < 14 (the reference group) [40]. Regarding PA intensities, two further outcomes of interest were binary variables for any mild PA and any PA of one or more intensities (mild, moderate, and/or strenuous) (henceforth termed ‘any PA’). For any mild PA, the non-reference group was a score > 0 for the mild component of the LSI, while the reference group was a score of 0 for the mild component of the LSI. For any PA, the non-reference group was a score > 0 for one or more of the mild, moderate, and strenuous components of the LSI, while the reference group was a score of 0 for all three components of the LSI. Any moderate PA and any strenuous PA were not assessed as separate outcomes due to excessively low numbers of participants who engaged in moderate and strenuous PA, 15 and 3, respectively.

### Other measures

Various demographic, medical, anthropometric, and energy-related characteristics were measured in the present study in order to describe the study population and/or include as independent variables in regression models assessing correlates of PA. Regarding demographic characteristics, age (years) was assessed as a continuous variable while gender (male/female), country of birth (overseas/Australia) and Aboriginal and/or Torres Strait Islander origin (yes/no) were defined as binary variables. Gender was not defined as a polytomous variable as no participants self-identified a non-binary gender. Regarding clinical variables, cancer type was assessed as a polytomous variable for descriptive purposes and a binary variable (breast cancer/non-breast cancer) for descriptive and regression purposes. The binary breast cancer variable was created because, firstly, most participants had breast cancer, secondly, a past study of PA correlates among rural cancer survivors dichotomised cancer type in the same way [12], and, thirdly, many past studies of PA correlates among cancer survivors were restricted to breast cancer survivors [11, 16, 17, 19–22, 26, 29]. We created a binary variable for treatment status (current anti-cancer treatment/completed or ceased anti-cancer treatment) because, firstly, some studies of PA correlates among cancer survivors defined cancer survivorship as the time from anti-cancer treatment rather than cancer diagnosis [18, 20, 22, 23] and, secondly, treatment types and complications have been shown to be associated with PA [20, 22]. Recruitment year was defined as a binary variable with categories of ‘2017–2019 (pre COVID-19 pandemic)’ and ‘2020–2021 (peri-COVID-19 pandemic)’. Regarding anthropometric variables, participants’ height and weight were used to calculate body mass index (BMI) in kg/m^2^. Participants’ BMI values were collapsed into a polytomous variable featuring the World Health Organization’s six BMI categories for descriptive purposes and a binary variable that categorised participants as presenting with obesity (BMI ≥ 30.0 kg/m^2^) or not (BMI < 30.0 kg/m^2^) for descriptive and regression purposes [41]. Lastly, participants’ energy levels were assessed because past studies found fatigue is associated with less PA among cancer survivors [12, 18, 26]. We assessed energy levels using a relevant item from a valid, reliable quality of life tool: the 7-item Functional Assessment of Cancer Therapy–General (FACT-G7) questionnaire [42]. This FACT-G7 item measured the extent to which participants lacked energy during the preceding week on a five-point Likert scale: not at all (0), a little bit (1), somewhat (2), quite a bit (3) and very much (4) [42]. In our study, participants’ energy levels were assessed in terms of a polytomous variable with all five energy categories for descriptive purposes, and a binary fatigue variable with categories of ‘yes’ (quite a bit or very much lacking in energy) and ‘no’ (not at all, a little bit or somewhat lacking in energy) for descriptive and regression purposes.

### Statistical analysis

Binary and polytomous variables were described using frequencies and percentages. Continuous variables were described using the median (lower quartile; upper quartile), mean (standard deviation), and minimum and maximum, as appropriate.

Binary logistic regression was used to assess associations between independent and outcome variables. The seven characteristics included as independent variables in binary logistic regression models were age (years) and five binary variables: gender, breast cancer, treatment status, recruitment year, obesity, and fatigue. These independent variables were chosen based on their inclusion in past studies that assessed PA correlates among cancer survivors [12, 14, 15, 17–20, 22–24, 26] and having adequate expected (as opposed to observed) cell counts ≥ 5 in two-way cross-tabulations with outcome variables (data not shown). Firstly, each of the seven independent variables was included in a separate univariable binary logistic regression model for each of three binary outcomes of interest: sufficient PA, any mild PA, and any PA. Subsequently, all seven independent variables were simultaneously included in a separate multivariable binary logistic regression model for each of the three binary outcomes. Crude odds ratios (cOR) and adjusted odds ratios (aORs) were calculated for univariable and multivariable models, respectively, alongside 95% confidence intervals (CIs) and p-values. Statistically significant associations were declared at the 5% level (*p*-values < 0.05).

Missing data were handled via complete-case analysis, whereby participants were only included in the sample if they had complete data for all variables. Statistical analyses were undertaken in Stata v15.0 (StataCorp, College Station, TX, USA).

### Ethics

This study was conducted in line with the Declaration of Helsinki (2013) and National Statement on Ethical Conduct in Human Research 2007 (Updated 2018). This study was approved by the West Gippsland Healthcare Group Research Ethics Committee (ID: ICAN), Latrobe Regional Hospital Human Research Ethics Committee (ID: 2020–14) and Monash University Human Research Ethics Committee (ID: 11890). All participants provided informed consent.

## Results

### Sample

Overall, 112 rural cancer survivors met the eligibility criteria. One hundred and three participants (92%) had complete data on all variables and were, thus, included in the study. Of the nine cancer survivors excluded due to missing data, four lacked anthropometric data and five lacked data on energy levels. Table 1 shows descriptive statistics for the 103 participants’ characteristics. Median and mean ages of participants were 66 and 64 years, respectively. Participants were mostly female (73%) and born in Australia (86%). All 103 participants reported that they were not of Aboriginal and/or Torres Strait Islander origin. The most common cancer type was breast (47%), followed by colorectal (20%) and then haematological (14%), and approximately half of the sample had completed or ceased anti-cancer treatment. Seventy-one per cent of participants were recruited before the onset of the COVID-19 pandemic in 2020. Forty-two per cent of participants presented with obesity, and 42% experienced fatigue.

**Table 1:** Demographic, medical, anthropometric, and energy-level characteristics of rural cancer survivors (*N* = 103)

Characteristic	*n* (column %)^†^
*Demographic*	
Age (years)	
Mean (SD)	64.3 (12.1)
Median (*Q*1; *Q*3)	65.7 (54.0; 73.5)
Gender	
Female	75 (72.8)
Male	28 (27.2)
Aboriginal and/or Torres Strait Islander origin	
No	103 (100.0)
Yes	0 (0)
Country of birth	
Australia	89 (86.4)
Country other than Australia	14 (13.6)
*Clinical*	
Cancer type	
Breast	48 (46.6)
Colorectal	21 (20.4)
Gynaecological	5 (4.9)
Haematological	14 (13.6)
Prostate	8 (7.8)
Other^‡^	7 (6.8)
Breast cancer	
Non-breast cancer	55 (53.4)
Breast cancer	48 (46.6)
Treatment status at baseline	
Current anti-cancer treatment	52 (50.5)
Completed/ceased anti-cancer treatment	51 (49.5)
Recruitment year	
2017–2019 (pre-COVID-19 pandemic)	73 (70.9)
2020–2021 (peri-COVID-19 pandemic)	30 (29.1)
*Anthropometric*	
BMI (kg/m^2^)	
Mean (SD)	29.4 (7.4)
Median (Q1; Q3)	28.0 (24.8; 32.6)
BMI group^§^	
Underweight (BMI < 18.5 kg/m^2^)	2 (1.9)
Normal weight (18.5 kg/m^2^ ≤ BMI < 25.0 kg/m^2^)	25 (24.3)
Pre-obesity (25.0 kg/m^2^ ≤ BMI < 30.0 kg/m^2^)	34 (33.0)
Obesity class I (30.0 kg/m^2^ ≤ BMI < 35.0 kg/m^2^)	25 (24.3)
Obesity class II (35.0 kg/m^2^ ≤ BMI < 40.0 kg/m^2^)	11 (10.7)
Obesity class III (40.0 kg/m^2^ ≥ BMI)	6 (5.8)
Obesity^§^	
No (BMI < 30 kg/m^2^)	61 (59.2)
Yes (BMI ≥ 30 kg/m^2^)	42 (40.8)
*Energy level*	
Lack of energy (FACT-G7 subscale)	
Not at all	2 (1.9)
A little bit	24 (23.3)
Somewhat	35 (34.0)
Quite a bit	23 (22.3)
Very much	19 (18.4)
Fatigue	
No^#^	61 (59.2)
Yes^††^	42 (40.8)

*SD* standard deviation, *Q1* lower quartile (25th percentile), *Q3* upper quartile (75th percentile), *n* frequency/numerator, *BMI* body mass index, *FACT-G7* 7-item Functional Assessment of Cancer Therapy–Gquestionnaire

^†^Unless otherwise specified in the ‘characteristic’ column

^‡^’Other’ (less frequently observed) cancers include lung, neuroendocrine, and upper gastrointestinal cancers as well as genitourinary cancers other than prostate cancer

^§^Classified using the World Health Organization’s definition

^#^A score of 0, 1, or 2 for the single item ‘I have a lack of energy’ from the 7-item Functional Assessment of Cancer Therapy–General (FACT-G7) questionnaire, measured on a scale of 0–4 where 0 = not at all, 1 = a little bit, 2 = somewhat, 3 = quite a bit, and 4 = very much

^††^A score of 3 or 4 for the single item ‘I have a lack of energy’ from the 7-item Functional Assessment of Cancer Therapy–General (FACT-G7) questionnaire, measured on a scale of 0–4 where 0 = not at all, 1 = a little bit, 2 = somewhat, 3 = quite a bit, and 4 = very much

### Physical activity

Table 2 describes participants’ levels of PA. Among the 103 participants, the median and mean LSI (total GSLTPAQ scores) were 6.0 and 10.1, respectively, on a scale of 0–73 for this sample. Overall, 35% of participants engaged in sufficient PA. Regarding PA intensities, 62% of participants engaged in any mild PA, 15% in any moderate PA, 3% in any strenuous PA, and 64% in any PA.

**Table 2:** Descriptive statistics for rural cancer survivors’ physical activity measured using the Leisure Score Index from the Godin–Shephard Leisure-Time Physical Activity Questionnaire (*N* = 103)

PA	Median (*Q*1; *Q*3)	Mean (SD)	Min.–Max	*n* (col. %)
*Item-specific scores*				
Mild PA	2.0 (0; 5.0)	2.4 (2.5)	0–9	-
Moderate PA	0 (0; 0)	0.5 (1.3)	0–6	-
Strenuous PA	0 (0; 0)	0.1 (0.4)	0–3	-
*Any PA by intensity*^†^				
Mild PA	-	-	-	64 (62.1)
Moderate PA	-	-	-	15 (14.6)
Strenuous PA	-	-	-	3 (2.9)
PA of one or more intensities	-	-	-	66 (64.1)
*LSI (total* GSLTPAQ* score)*	6.0 (0; 15.0)	10.1 (12.1)	0–73	-
*Sufficient activity*^‡^	-	-	-	36 (35.0)

*PA* physical activity, *Q1* lower quartile (25th percentile), *Q3* upper quartile (75th percentile), *SD* standard deviation, *min*. minimum, *max*. maximum, *n* frequency/numerator, *LSI* Leisure Score Index, *GSLTPAQ* Godin–Shephard Leisure-Time Physical Activity Questionnaire

^†^Leisure score index > 0 for a given item-specific score

^‡^Leisure score index ≥ 14 for the total score

### Correlates of physical activity

Regarding the primary PA outcome, obesity and fatigue were each independently associated with significantly lower odds of having sufficient PA (Table 3). Participants who presented with obesity had 79% lower odds of having sufficient PA (aOR = 0.21, 95% CI = 0.07–0.61) while participants who experienced fatigue had 70% lower odds of having sufficient PA (aOR = 0.30, 95% CI = 0.11–0.83). Age, gender, cancer type, treatment status, and recruitment year were unrelated to sufficient PA (Table 3).

**Table 3:** Descriptive and binary logistic regression results for rural cancer survivors’ characteristics associated with sufficient physical activity, measured using the Leisure Score Index from the Godin–Shephard Leisure-Time Physical Activity Questionnaire (*N* = 103)

	Leisure Score Index		Sufficiently active (Leisure Score Index ≥ 14)		
Characteristic	Median (*Q*1; *Q*3)	Mean (SD)^†^	*n* (row %)^†^	cOR (95% CI)	aOR (95% CI)^‡^
*Demographic*					
Age (years) (*N* = 103)	*r* = − 0.04	*r* = − 0.04	M (SD): 65.3 (12.6)	1.01 (0.98–1.05)	0.98 (0.94–1.02)
Gender					
Female (*N* = 75)	9.0 (0; 15.0)	10.9 (12.7)	28 (37.3)	1.00	1.00
Male (*N* = 28)	4.5 (0; 15.0)	8.0 (10.0)	8 (28.6)	0.67 (0.26–1.73)	0.36 (0.10–1.28)
Country of birth					
Australia (*N* = 89)	6.0 (0; 15.0)	9.9 (12.0)	30 (33.7)	-	-
Country other than Australia (*N* = 14)	6.0 (0; 21.0)	11.9 (13.0)	6 (42.9)	-	-
*Clinical*					
Cancer type					
Breast (*N* = 48)	6.0 (0; 15.0)	8.6 (10.0)	14 (29.2)	-	-
Colorectal (*N* = 21)	9.0 (0; 21.0)	13.3 (13.1)	10 (47.6)	-	-
Gynaecological (*N* = 5)	15.0 (12.0; 19.0)	17.2 (10.5)	3 (60.0)	-	-
Haematological (*N* = 14)	6.0 (0; 15.0)	11.2 (19.1)	4 (28.6)	-	-
Prostate (*N* = 8)	6.0 (0; 15.0)	7.9 (8.2)	3 (37.5)	-	-
Other^§^ (*N* = 7)	3.0 (0; 15.0)	6.4 (8.2)	2 (28.6)	-	-
Breast cancer					
Non-breast cancer (*N* = 55)	6.0 (0; 15.0)	11.5 (13.6)	22 (40.0)	1.00	1.00
Breast cancer (*N* = 48)	6.0 (0; 15.0)	8.6 (10.0)	14 (29.2)	0.62 (0.27–1.41)	0.36 (0.12–1.10)
Treatment status at baseline					
Current anti-cancer treatment (*N* = 52)	6.0 (0; 15.0)	9.3 (13.1)	14 (26.9)	1.00	1.00
Completed/ceased anti-cancer treatment (*N* = 51)	9.0 (0; 15.0)	10.9 (11.0)	22 (43.1)	2.06 (0.90–4.70)	1.87 (0.72–4.81)
Recruitment year					
2017–2019 (pre-COVID-19 pandemic) (*N* = 73)	9.0 (0; 15.0)	11.2 (13.2)	28 (38.4)	1.00	1.00
2020–2021 (peri-COVID-19 pandemic) (*N* = 30)	6.0 (0; 15.0)	7.5 (8.5)	8 (26.7)	0.58 (0.23–1.49)	0.55 (0.18–1.63)
*Anthropometric*					
BMI (kg/m^2^)	*r* = − 0.23	*r* = − 0.23	M (SD): 26.8 (4.9)	-	-
BMI group^¶^					
Underweight (BMI < 18.5 kg/m^2^) (*N* = 2)	10.5 (0; 21.0)	10.5 (14.8)	1 (50.0)	-	-
Normal weight (18.5 kg/m^2^ ≤ BMI < 25.0 kg/m^2^) (*N* = 25)	9.0 (0; 21.0)	12.1 (11.0)	12 (48.0)	-	-
Pre-obesity (25.0 kg/m^2^ ≤ BMI < 30.0 kg/m^2^) (*N* = 34)	12.0 (3.0; 21.0)	13.3 (11.5)	16 (47.1)	-	-
Obesity class I (30.0 kg/m^2^ ≤ BMI < 35.0 kg/m^2^) (*N* = 25)	3.0 (0; 12.0)	8.8 (15.2)	5 (20.0)	-	-
Obesity class II (35.0 kg/m^2^ ≤ BMI < 40.0 kg/m^2^) (*N* = 11)	0 (0; 0)	1.9 (4.7)	1 (9.1)	-	-
Obesity class III (40.0 kg/m^2^ ≥ BMI) (*N* = 6)	3.0 (0; 6.0)	4.5 (5.9)	1 (16.7)	-	-
Obesity^¶^					
No (BMI < 30 kg/m^2^) (*N* = 61)	12.0 (3.0; 21.0)	12.7 (11.2)	29 (47.5)	1.00	1.00
Yes (BMI ≥ 30 kg/m^2^) (*N* = 42)	0 (0; 10.0)	6.4 (12.4)	7 (16.7)	0.22 (0.08–0.57)*	0.21 (0.07–0.61)*
*Energy level*					
Lack of energy (FACT-G7 subscale)					
Not at all (*N* = 2)	10.5 (0; 21.0)	10.5 (14.8)	1 (50.0)	-	-
A little bit (*N* = 24)	15.0 (6; 22.5)	16.1 (12.1)	15 (62.5)	-	-
Somewhat (*N* = 35)	6.0 (0; 15.0)	9.1 (9.7)	12 (34.3)	-	-
Quite a bit (*N* = 23)	6.0 (0; 12.0)	7.0 (8.8)	4 (17.4)	-	-
Very much (*N* = 19)	0 (0; 12.0)	8.3 (17.0)	4 (21.1)	-	-
Fatigue					
No^#^ (*N* = 61)	10.0 (0; 21.0)	11.9 (11.2)	28 (45.9)	1.00	1.00
Yes^††^ (*N* = 42)	3.0 (0; 12.0)	7.6 (13.0)	8 (19.0)	0.28 (0.11–0.70)*	0.30 (0.11–0.83)*

*Q1* lower quartile (25 th percentile), *Q3* upper quartile (75 th percentile), *SD* standard deviation, *n* frequency/numerator, *cOR* crude odds ratio, *CI* confidence interval, *aOR* adjusted odds ratio, *r* correlation coefficient, *N* sample size/denominator, *BMI* body mass index, *FACT-G7* 7-item Functional Assessment of Cancer Therapy–General questionnaire

^†^Unless otherwise specified

^‡^Adjusted for all other variables included in the multivariable binary logistic regression model (i.e., all variables with an aOR value in this table)

^§^ ‘Other’ (i.e., non-breast) cancers include colorectal, genitourinary, gynaecological, haematological, lung, neuroendocrine, and upper gastrointestinal cancers

^¶^Classified using the World Health Organization’s definition

^#^A score of 0, 1, or 2 for the single item ‘I have a lack of energy’ from the 7-item Functional Assessment of Cancer Therapy–General (FACT-G7) questionnaire, measured on a scale of 0–4 where 0 = not at all, 1 = a little bit, 2 = somewhat, 3 = quite a bit, and 4 = very much

†† A score of 3 or 4 for the single item ‘I have a lack of energy’ from the 7-item Functional Assessment of Cancer Therapy–General (FACT-G7) questionnaire, measured on a scale of 0–4 where 0 = not at all, 1 = a little bit, 2 = somewhat, 3 = quite a bit, and 4 = very much

^*^Statistically significant at the 5% level (*p*-value < 0.05)

Regarding PA intensity outcomes, breast cancer was independently associated with significantly lower odds of any mild PA, and obesity was independently associated with significantly lower odds of any mild PA and any PA (Table 4). Participants with breast cancer had 73% (aOR = 0.27, 95% CI = 0.08–0.87) lower odds of any mild PA, while participants who presented with obesity had 74% (aOR = 0.26, 95% CI = 0.10–0.66) and 74% (aOR = 0.26, 95% CI = 0.10–0.64) lower odds of any mild PA and any PA, respectively. Breast cancer was unrelated to any PA, while age, gender, treatment status, recruitment year, and fatigue were unrelated to any mild PA and any PA (Table 4).

**Table 4:** Descriptive and binary logistic regression results for rural cancer survivors’ characteristics associated with any mild physical activity and any physical activity of one or more intensities, measured using the Leisure Score Index from the Godin–Shephard Leisure-Time Physical Activity Questionnaire (*N* = 103)

	Any mild PA (item-specific score > 0)			Any PA (item-specific score > 0) of one or more intensities		
Characteristic	*n* (row %)^†^	cOR (95% CI)	aOR (95% CI)^‡^	*n* (row %)^†^	cOR (95% CI)	aOR (95% CI)^‡^
*Demographic*						
Age (years) (*N* = 64 mild PA; 66 any PA)	*M* (*Q*1; *Q*3): 68.2 (57.1; 74.6)	1.03 (0.99–1.06)	1.01 (0.97–1.06)	*M* (*Q*1; *Q*3): 66.9 (54.0; 74.6)	1.02 (0.99–1.05)	1.00 (0.96–1.05)
Gender						
Female (*N* = 75)	47 (62.7)	1.00	1.00	49 (65.3)	1.00	1.00
Male (*N* = 28)	17 (60.7)	0.92 (0.38–2.24)	0.28 (0.07–1.10)	17 (60.7)	0.82 (0.34–2.01)	0.31 (0.08–1.18)
Country of birth						
Australia (*N* = 89)	54 (60.7)	-	-	56 (62.9)	-	-
Country other than Australia (*N* = 14)	10 (71.4)	-	-	10 (71.4)	-	-
*Clinical*						
Cancer type						
Breast (*N* = 48)	25 (52.1)	-	-	27 (56.3)	-	-
Colorectal (*N* = 21)	15 (71.4)	-	-	15 (71.4)	-	-
Gynaecological (*N* = 5)	5 (100.0)	-	-	5 (100.0)	-	-
Haematological (*N* = 14)	9 (64.3)	-	-	9 (64.3)	-	-
Prostate (*N* = 8)	5 (62.5)	-	-	5 (62.5)	-	-
Other^§^ (*N* = 7)	5 (71.4)	-	-	5 (71.4)	-	-
Breast cancer						
Non-breast cancer (*N* = 55)	39 (70.9)	1.00	1.00	39 (70.9)	1.00	1.00
Breast cancer (*N* = 48)	25 (52.1)	0.45 (0.20–1.00)	0.27 (0.08–0.87)*	27 (56.3)	0.53 (0.23–1.19)	0.32 (0.10–1.03)
Treatment status at baseline						
Current anti-cancer treatment (*N* = 52)	32 (61.5)	1.00	1.00	32 (61.5)	1.00	1.00
Completed/ceased anti-cancer treatment (*N* = 51)	32 (62.7)	1.05 (0.47–2.33)	0.85 (0.34–2.12)	34 (66.7)	1.25 (0.56–2.80)	1.05 (0.42–2.60)
Recruitment year						
2017–2019 (pre-COVID-19 pandemic) (*N* = 73)	48 (65.8)	1.00	1.00	49 (67.1)	1.00	1.00
2020–2021 (peri-COVID-19 pandemic) (*N* = 30)	16 (53.3)	0.60 (0.25–1.41)	0.69 (0.26–1.84)	17 (56.7)	0.64 (0.27–1.53)	0.73 (0.27–1.93)
*Anthropometric*						
BMI (kg/m^2^) (*N* = 64 mild PA; 66 any PA)	*M* (*Q*1; *Q*3): 27.1 (23.8; 30.7)	-	-	*M* (*Q*1; *Q*3): 27.2 (24.0; 31.1)	-	-
BMI group^¶^						
Underweight (BMI < 18.5 kg/m^2^) (*N* = 2)	1 (50.0)	-	-	1 (50.0)	-	-
Normal weight (18.5 kg/m^2^ ≤ BMI < 25.0 kg/m^2^) (*N* = 25)	18 (72.0)	-	-	18 (72.0)	-	-
Pre-obesity (25.0 kg/m^2^ ≤ BMI < 30.0 kg/m^2^) (*N* = 34)	27 (79.4)	-	-	28 (82.4)	-	-
Obesity class I (30.0 kg/m^2^ ≤ BMI < 35.0 kg/m^2^) (*N* = 25)	13 (52.0)	-	-	14 (56.0)	-	-
Obesity class II (35.0 kg/m^2^ ≤ BMI < 40.0 kg/m^2^) (*N* = 11)	2 (18.2)	-	-	2 (18.2)	-	-
Obesity class III (40.0 kg/m^2^ ≥ BMI) (*N* = 6)	3 (50.0)	-	-	3 (50.0)	-	-
Obesity^¶^						
No (BMI < 30 kg/m^2^) (*N* = 61)	46 (75.4)	1.00	1.00	47 (77.0)	1.00	1.00
Yes (BMI ≥ 30 kg/m^2^) (*N* = 42)	18 (42.9)	0.24 (0.11–0.57)*	0.26 (0.10–0.66)*	19 (45.2)	0.25 (0.10–0.58)*	0.26 (0.10–0.64)*
*Energy level*						
Lack of energy (FACT-G7 subscale)						
Not at all (*N* = 2)	1 (50.0)	-	-	1 (50.0)	-	-
A little bit (*N* = 24)	20 (83.3)	-	-	21 (87.5)	-	-
Somewhat (*N* = 35)	22 (62.9)	-	-	22 (62.9)	-	-
Quite a bit (*N* = 23)	12 (52.2)	-	-	13 (56.5)	-	-
Very much (*N* = 19)	9 (47.4)	-	-	9 (47.4)	-	-
Fatigue^#^						
No^#^ (*N* = 61)	43 (70.5)	1.00	1.00	44 (72.1)	1.00	1.00
Yes^††^ (*N* = 42)	21 (50.0)	0.42 (0.18–0.95)*	0.41 (0.16–1.04)	22 (52.4)	0.43 (0.19–0.97)*	0.43 (0.17–1.09)

*PA* physical activity, *M* median, *Q1* lower quartile (25th percentile), *Q3* upper quartile (75th percentile), *n* frequency/numerator, *cOR* crude odds ratio, *CI* confidence interval, *aOR* adjusted odds ratio, *r* correlation coefficient, *N* sample size/denominator, *BMI* body mass index, FACT-G7 – 7-item Functional Assessment of Cancer Therapy – General questionnaire

^†^Unless otherwise specified

^‡^Adjusted for all other variables included in the multivariable binary logistic regression model (i.e., all variables with an aOR value in this table)

^§^ ‘Other’ (i.e., non-breast) cancers include colorectal, genitourinary, gynaecological, haematological, lung, neuroendocrine, and upper gastrointestinal cancers

^¶^Classified using the World Health Organization’s definition

^#^A score of 0, 1, or 2 for the single item ‘I have a lack of energy’ from the 7-item Functional Assessment of Cancer Therapy–General (FACT-G7) questionnaire, measured on a scale of 0–4 where 0 = not at all, 1 = a little bit, 2 = somewhat, 3 = quite a bit, and 4 = very much

^††^A score 3 or 4 for the single item ‘I have a lack of energy’ from the 7-item Functional Assessment of Cancer Therapy–General (FACT-G7) questionnaire, measured on a scale of 0–4 where 0 = not at all, 1 = a little bit, 2 = somewhat, 3 = quite a bit, and 4 = very much

^*^Statistically significant at the 5% level (*p*-value < 0.05)

## Discussion

In this cross-sectional study set in rural Victoria, Australia, 35% of 103 cancer survivors had sufficient PA, 62% had any mild PA, and 64% had any PA. Few participants reported any moderate (15%) or strenuous (3%) PA. The proportion of cancer survivors with sufficient PA in our study is comparable to the proportions reported for 4,295 rural cancer survivors in South Australia (40%) [43] and for 219 rural cancer survivors in Pennsylvania (41%) [25]. No known studies have reported proportions of rural cancer survivors who engaged in PA of different intensities, suggesting we have presented novel PA statistics for future benchmarking.

Regarding PA correlates among rural Australian cancer survivors, we found that obesity was associated with lower odds of all three outcomes: sufficient PA, any mild PA, and any PA. There is evidence to suggest that the relationship between obesity and PA in the general population is bidirectional, with lower PA contributing to obesity and obesity contributing to lower PA [44]. In a cross-sectional study conducted among Australian and Canadian colon cancer survivors, obesity was independently associated with significantly less mild PA [23]. Additionally, in cross-sectional studies conducted among lung cancer survivors, increasing BMI was independently associated with less mild PA and less moderate PA in Canadian and Chinese contexts, respectively [14, 15]. Increasing BMI was also found to be independently associated with significantly less vigorous PA among US breast cancer survivors [29]. Ours is the first known study to find such associations specifically among rural cancer survivors.

Another key finding of our study was the significantly lower odds of sufficient PA for rural cancer survivors experiencing fatigue. This association may also be bidirectional, as suggested by a study revealing a bidirectional relationship between colorectal cancer-related fatigue (CRF) and PA [45]. While CRF may reduce people’s inclination or capacity for PA, PA has somewhat paradoxically been found to improve CRF in meta-analyses [46, 47]. Organisations such as the American Society of Clinical Oncology and Clinical Oncology Society of Australia recommend PA and exercise in the management of CRF [7, 8]. Thus, rural cancer survivors experiencing fatigue could be educated on the beneficial effects of PA on CRF and prioritised for exercise interventions. Previously, fatigue was independently associated with less moderate and strenuous PA among metropolitan breast cancer survivors in Indonesia and less moderate-intensity PA among rural breast cancer survivors in the US [18, 26]. We were unable to assess the relationship between fatigue and each of moderate and strenuous PA (separately) in our study due to excessively low numbers of participants who engaged in PA of these intensities. Fatigue was associated with significantly less sufficient PA among survivors of any cancer type in rural Canada, although this association was unadjusted for potential confounders [12]. Ours is the first known study to find that fatigue is independently associated with less sufficient PA among rural cancer survivors.

The lower odds of any mild PA for participants diagnosed with breast rather than non-breast cancer are a further novel finding. While the explanation for this association is unclear and warrants further investigation, the associations may be explained by the nature of breast cancer and/or its specific treatment options: surgery followed by chemotherapy, radiation therapy, and/or hormonal therapy. No past studies have reported associations between a breast cancer diagnosis and intensity/ies of PA among rural and/or metropolitan cancer survivors. Interestingly, in the present study, breast cancer was independently associated with lower odds of any mild PA yet unrelated to any PA and sufficient PA. All three aORs for breast cancer were, however, of similar magnitudes—0.27, 0.32, and 0.36 for the associations with any mild PA, any PA, and sufficient PA, respectively. A Canadian cross-sectional study also found that a diagnosis of breast rather than non-breast cancer was unrelated to sufficient PA among rural cancer survivors, albeit without adjustment for potential confounders [12]. Further studies of associations between breast cancer and PA are required.

While certain treatment types have been found to be associated with PA in past studies (e.g., chemotherapy and/or radiation therapy were associated with reduced PA in German breast cancer survivors [22]), we found no statistically significant difference in PA between those cancer survivors who were currently receiving anti-cancer treatment and those cancer survivors who had completed or ceased anti-cancer treatment. Nevertheless, the proportion of rural Australian cancer survivors with sufficient PA was at 16 percentage points greater among those who had completed/ceased treatment (43%) than those who were currently receiving treatment (27%) (Table 3). As the 95% CIs surrounding the cOR and aOR for the treatment status variable were wide (Table 3), the lack of statistical significance could be due to our study’s low sample size of 103.

Our study has three key strengths: use of valid and reliable measures of PA and energy levels [39, 42], use of an evidence-based approach to selecting independent variables for regression analyses, and little chance of selection bias as only 8% of participants were excluded due to missing data.

Our study also has limitations. Consistent with most studies in this research area [11–20, 24–28], our study was limited by the use of a cross-sectional design and, thus, unknown directionality of associations. Furthermore, while resistance activities are a subtype of PA recommended for cancer survivors, the GSLTPAQ does not specifically measure resistance activities [8, 38]. Data on participant-reported measures (i.e., the GSLTPAQ and the energy level item from the FACT-G7 questionnaire) could have been affected by recall bias. There may have been residual confounding by factors associated with both independent and outcome variables that were not controlled for in multivariable binary logistic regression models (e.g., comorbidity score [26] and cancer stage [18]). Sample sizes larger than 103 would be needed to accommodate additional independent variables in multivariable binary logistic regression models. Nevertheless, in our study, confounding by cancer stage was partially controlled by design through restriction to rural cancer survivors who were not receiving end-of-life care. The CIs around effect estimates were wide in our study because, in line with the small rural hospital setting, the sample size was 103. Lastly, as we employed convenience sampling in one rural Australian hospital, our results may only be generalisable to populations of rural cancer survivors with similar characteristics to our study population.

## Conclusion

We found that, among 103 cancer survivors receiving care at a rural Australian hospital, rates of sufficient PA, mild PA, and any PA were 35%, 62%, and 64%, respectively. Rural cancer survivors with fatigue had lower odds of sufficient PA, rural cancer survivors with obesity had lower odds of sufficient PA, any mild PA, and any PA, and rural breast cancer survivors had lower odds of any mild PA. Further studies are needed to confirm and build upon our study’s findings, preferably in larger samples and using the cohort study design. Nevertheless, our results suggest that, in this and similar rural settings, oncology referrals to exercise physiologists and exercise interventions such as health coaching could be preferentially offered to the substantial proportion—approximately two-thirds—of cancer survivors with insufficient PA. In particular, these interventions could be tailored to and targeted towards those rural cancer survivors with obesity, fatigue, and/or breast cancer. This tailored and targeted approach to exercise oncology could help to streamline delivery of supportive cancer care in under-resourced rural settings.

## Data Availability

The dataset underlying the present study’s findings is not publicly available due to ethical/privacy restrictions on the availability of data. The dataset is, however, available from the corresponding author upon reasonable request.
